# Metabolic Changes During a Calorie‐Restricted, Fish‐Rich Ketogenic Diet Associated With Increased Circulating TMAO in Healthy Adults

**DOI:** 10.1002/edm2.70360

**Published:** 2026-10-16

**Authors:** Katarzyna Siedzik, Kamil Góral, Damian Flis, Maciej Jaguszewski, Robert A. Olek, Wiesław Ziółkowski

**Affiliations:** ^1^ Doctoral School Poznan University of Physical Education Poznan Poland; ^2^ Department of Pharmaceutical Pathophysiology, Faculty of Pharmacy Medical University of Gdansk Gdansk Poland; ^3^ Calculation Centre Poznan University of Physical Education Poznan Poland; ^4^ Department of Athletics, Strength, and Conditioning Poznan University of Physical Education Poznan Poland; ^5^ Department of Rehabilitation Medicine, Faculty of Health Sciences Medical University of Gdansk Gdansk Poland

**Keywords:** blood lipids, carnitine, glycemic management, insulin, ketogenic diet, leptin, resistin, trimethylamine N‐oxide

## Abstract

**Background:**

A ketogenic diet (KD) is increasingly investigated as a nutritional approach for improving glycemic control and metabolic health. However, relatively few studies have examined fish‐rich ketogenic interventions. Fish consumption may substantially increase circulating trimethylamine N‐oxide (TMAO), while habitual fish intake is generally associated with favourable cardiometabolic outcomes. The metabolic significance of a short‐term diet‐induced increase in TMAO therefore remains unclear. This study aimed to characterize changes in circulating TMAO and related metabolites, glycemic and insulin‐related biomarkers, oxidative stress markers, and lipid profiles during a fish‐rich, calorie‐restricted KD in healthy adults.

**Methods:**

Nine men and five women (mean age 45 ± 3 years) participated in a 2‐week, single‐arm, within‐subject pre–post dietary intervention. TMAO and related metabolites, selected glycemic and insulin‐related biomarkers, oxidative stress markers, and lipid‐profile parameters were assessed before and after the intervention. Participants followed individualized fish‐rich ketogenic meal plans providing an approximately 500 kcal/day energy deficit.

**Results:**

Following the intervention, circulating TMAO and betaine concentrations increased significantly, whereas carnitine and glycerophosphocholine (GPC) concentrations decreased. Favourable changes were observed in fasting glucose, insulin, C‐peptide, leptin, and resistin. HDL cholesterol decreased significantly, while total cholesterol, LDL cholesterol, and triglycerides remained unchanged. No evidence of increased oxidative stress was observed. The direct estimate of insulin sensitivity (HOMA‐%S) did not reach statistical significance after correction for multiple comparisons.

**Conclusions:**

A 2‐week, calorie‐restricted, fish‐rich ketogenic intervention was associated with a marked increase in circulating TMAO together with favourable changes in selected glycemic, insulin‐related, and adipokine biomarkers, without evidence of increased oxidative stress. HDL cholesterol decreased, whereas other lipid‐profile parameters remained largely unchanged. Because caloric restriction, ketogenic macronutrient composition, high fish intake, and reductions in body weight and adiposity occurred simultaneously, the independent contribution of TMAO to these metabolic changes cannot be determined. These findings should therefore be considered preliminary and hypothesis‐generating.

## Graphical abstract

A 2‐week calorie‐restricted, fish‐rich ketogenic diet increased circulating TMAO while improving selected glycemic and insulin‐related biomarkers in healthy adults. HDL cholesterol decreased, whereas oxidative stress markers remained unchanged, highlighting a complex short‐term metabolic response that should not be interpreted as evidence of causality.

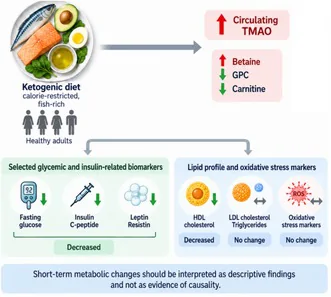

## 1 Introduction

In recent years, increasing attention has been directed toward the interactions among diet, the gut microbiota, and host metabolic health. Microbial metabolites derived from dietary components may contribute to the regulation of cardiometabolic processes [1]. Among them, trimethylamine N‐oxide (TMAO) has emerged as a potential link between dietary exposure and metabolic dysfunction [2, 3]. Elevated circulating TMAO concentrations have been associated with cardiovascular disease and other chronic conditions [4], possibly through mechanisms involving altered cholesterol metabolism, foam cell formation, endothelial dysfunction, and inflammation [5, 6]. However, whether TMAO is directly involved in disease development or primarily reflects dietary intake, microbial activity, renal function, and underlying metabolic status remains uncertain.

TMAO has also been associated with disturbances in glucose homeostasis, insulin sensitivity, and lipid metabolism [7, 8, 9]. A meta‐analysis of 12 clinical studies reported a dose–response relationship between circulating TMAO and diabetes risk, with a 54% increase in risk for each 5 μM increment in plasma TMAO [8]. Nevertheless, most available evidence is observational and is derived largely from individuals with established disease or elevated cardiometabolic risk. Consequently, the metabolic significance of increased TMAO concentrations in otherwise healthy individuals remains incompletely understood.

TMAO can originate from both endogenous metabolism and direct dietary intake. In the endogenous pathway, intestinal microorganisms convert dietary precursors, including choline, phosphatidylcholine, L‐carnitine, and betaine, into trimethylamine (TMA). TMA is subsequently absorbed and oxidized in the liver, predominantly by flavin‐containing monooxygenase 3 (FMO3), to form TMAO [10] (Figure 1). These precursors are widely distributed in food: choline and phosphatidylcholine occur primarily in eggs, meat, dairy products, and some fish; L‐carnitine is particularly abundant in red meat; and betaine is supplied mainly by plant foods such as whole grains, spinach, and beets [11, 12].

**FIGURE 1:**
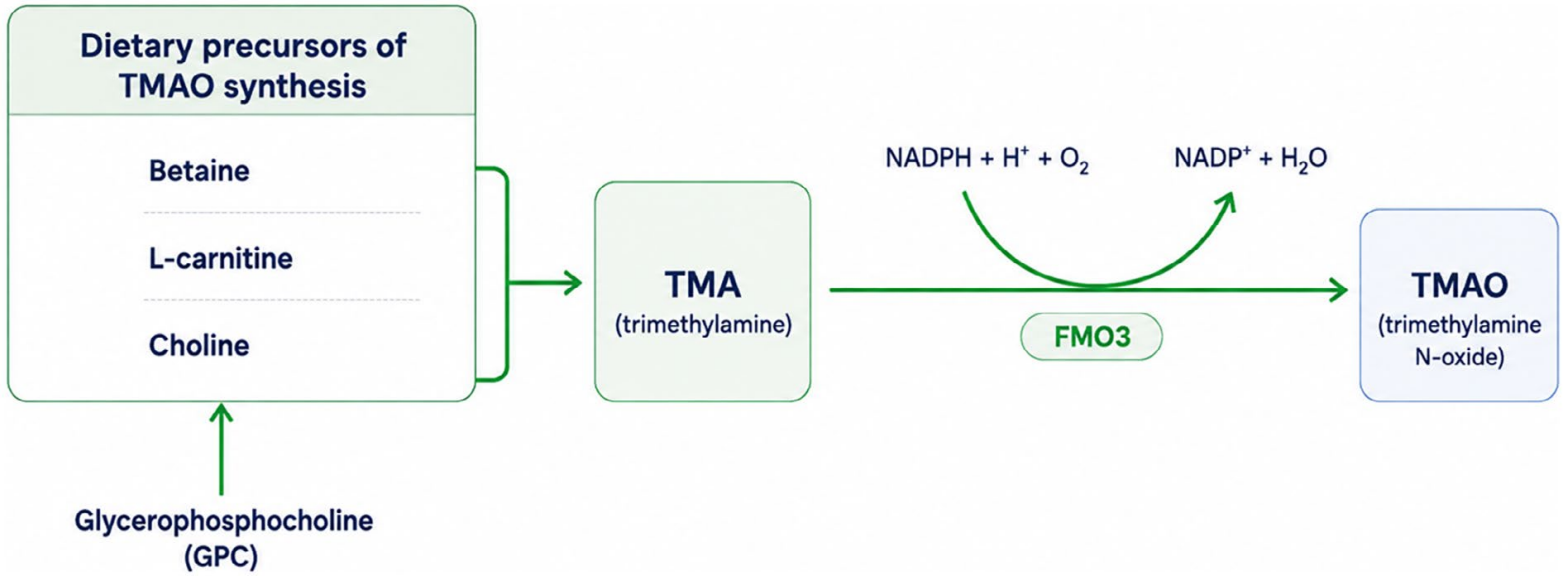
Scheme showing the synthesis of TMAO.

Fish and seafood represent an important exception because they contain substantial amounts of preformed TMAO that can be absorbed independently of gut microbial conversion [13, 14]. Accordingly, fish intake may induce a pronounced postprandial increase in circulating TMAO, with concentrations reported to reach approximately 150 μM within 2 h of ingestion [15]. However, habitual fish consumption, particularly the consumption of fatty fish, is generally associated with favourable cardiovascular outcomes. A recent meta‐analysis of prospective cohort studies reported an approximately 8% lower cardiovascular risk when comparing high with low fish consumption, while fatty fish intake has been associated with lower coronary heart disease incidence and mortality [16]. Thus, an increase in circulating TMAO following fish consumption should not necessarily be interpreted as evidence of an adverse dietary effect.

The potential relationship between TMAO and redox homeostasis adds further complexity. Oxidative stress contributes to the development of disorders of glucose and lipid metabolism [17], and TMAO may participate in redox reactions involving protein sulfhydryl groups and low‐molecular‐weight thiols, including glutathione [18]. Conversely, experimental evidence indicates that TMAO may, under certain conditions, enhance antioxidant defence through activation of the nuclear factor erythroid 2‐related factor 2 (Nrf2) pathway [19]. These apparently divergent effects suggest that the biological consequences of elevated TMAO may depend on its dietary source, metabolic context, and duration of exposure.

A short‐term, energy‐restricted, fish‐rich ketogenic diet provides a useful model for examining this issue because it combines a high intake of fish‐derived TMAO and its precursors with marked changes in substrate utilization and energy balance. However, the concurrent effects of preformed TMAO exposure, carbohydrate restriction, and negative energy balance on circulating TMAO, metabolic outcomes, and redox status in healthy adults remain poorly characterized.

Therefore, this study aimed to characterize changes in circulating TMAO and related metabolites, glycemic parameters, lipid profile, body composition, and selected markers of redox status during a 2‐week, energy‐restricted, fish‐rich ketogenic dietary intervention in healthy adults. We additionally explored whether changes in TMAO concentrations were associated with concurrent changes in metabolic and redox‐related parameters.

## 2 Material and Methods

### 2.1 Ethics

Committee for Scientific Research (KB 27‐19) and was registered in the ClinicalTrials.gov registry (registration number NCT05558488). All participants were informed about the basic principles of KD, including allowed and prohibited products, as well as the risks and expected outcomes, before starting the experimental procedures. Written informed consent for participation was obtained.

### 2.2 Subjects

Fourteen participants (nine men and five women; mean age, 45 ± 3 years; height, 174 ± 10 cm; body mass, 76.7 ± 11.1 kg) were recruited through self‐selection. Initially, 20 individuals volunteered to participate, of whom 14 met the eligibility criteria and were enrolled in the study. Eligible participants were healthy adult non‐smokers. Exclusion criteria included cardiovascular, thyroid, gastrointestinal, respiratory, or other metabolic diseases; adherence to special diets; use of dietary supplements; and use of medications affecting blood lipid or glucose concentrations. Before the baseline examination, participants were instructed to avoid alcohol consumption and physical exercise for at least 48 h. They were also asked to suspend their regular training schedule throughout the 14‐day intervention period.

For the correlation analysis between TMAO concentrations and selected indicators of glycemic and metabolic health, only participants who met the following criteria were included: (1) an increase in TMAO concentration after the KD intervention, and (2) a baseline TMAO concentration within the mean ± 3 SD range. One participant did not meet both criteria and was therefore excluded from the correlation analyses.

### 2.3 Study Design

The study was designed as a 2‐week, single‐arm, within‐subject pre–post dietary intervention in healthy adults. During the intervention, participants followed an individually energy‐adjusted, fish‐rich ketogenic diet providing an approximately 500 kcal/day energy deficit.

The present study represents a secondary analysis of the same 14‐participant dietary intervention cohort previously reported by Siedzik et al. [20]. The previous publication focused on body composition, skeletal muscle mass and strength, somatic symptoms, and nutritional ketosis. Body‐composition measures (body mass, fat mass, fat‐free mass, and visceral adipose tissue) and blood and urinary ketone measurements overlap between the two publications and are included here to characterize the intervention and provide metabolic context. In contrast, circulating TMAO and related metabolites (including choline, betaine, and carnitine), glycemic and insulin‐related markers, lipid‐profile parameters, redox‐related markers, and analyses of their associations with TMAO were not reported in the previous publication and constitute the primary novel outcomes of the present study. Isometric muscle strength and somatic symptom outcomes reported previously are not analysed in the present manuscript.

However, the current manuscript addresses a different research question and presents novel findings not included in the previous publication. Previously reported pre‐ and post‐intervention values for selected overlapping variables were used to calculate change scores (Δ values), which were subsequently included in exploratory analyses examining associations with changes in TMAO and other metabolic or redox‐related parameters. Specifically, previously reported body‐composition measurements were used to derive Δbody fat, Δbody fat percentage, and Δvisceral adipose tissue. These change scores were used in the present study for exploratory association analyses that were not performed or reported in our previous publication [20].

Prior to the commencement of the study, the participants met with the principal investigator and were thoroughly instructed on the study's design, the implementation of the KD, and the monitoring of any symptoms. Anthropometric and performance measurements were conducted 1 day prior to the intervention and subsequently after 14 days. This 2‐week timeframe is considered sufficient to capture the initial physiological adaptations associated with a KD, including reductions in total body mass and visceral adipose tissue, which are commonly observed within the first 14 days of dietary adherence. Given the study's emphasis on short‐term responses, the selected duration is appropriate for evaluating rapid alterations in body composition. Furthermore, the minimal decrease in skeletal muscle mass alongside the preservation of isometric muscle strength supports the adequacy of this period for assessing early‐stage outcomes, particularly in athletic populations seeking expedited performance or aesthetic benefits. The measurements were performed under the same conditions and by the same operators.

### 2.4 Dietary Intervention

For each participant, the estimated energy requirements were calculated using the Mifflin–St Jeor equation for the basal metabolic rate [21], adjusted for physical activity levels assessed from 7‐day activity records. The basal metabolic rate served as the basis for developing individualized nutritional plans incorporating a daily energy deficit of 500 kcal. The detailed methodology has been described elsewhere [20].

Protein intake was approximately 1.6 g/kg body weight/day, corresponding to approximately 20% of total energy intake, while approximately 75%–76% of energy was derived from fat. Carbohydrate intake was restricted to < 30 g/day. The intervention included cod, mackerel, pollock, tuna, salmon, and trout, together with vegetables, eggs, low‐glycemic‐index fruits, plant oils, avocado, and olives. For the representative 2000‐kcal dietary plan, the macronutrient distribution was 20.2% protein, 76.4% fat, and 3.4% carbohydrate.

The n‐3 PUFA content of the intervention was estimated from the quantities and types of foods specified in the meal plans using USDA food composition data. The representative 2000‐kcal meal plan contained an average of approximately 112.6 g/day of flaxseed oil and 288.4 g/day of fish. α‐Linolenic acid (ALA; 18:3 n‐3) was supplied predominantly by flaxseed oil, whereas the principal long‐chain marine n‐3 PUFAs, eicosapentaenoic acid (EPA; 20:5 n‐3) and docosahexaenoic acid (DHA; 22:6 n‐3), were supplied primarily by fish. Estimated ALA intake from flaxseed oil was approximately 55.1 g/day, while estimated EPA and DHA intakes from fish were approximately 0.69 and 1.70 g/day, respectively (EPA + DHA: approximately 2.39 g/day). Individual meal plans followed the same qualitative dietary pattern but were quantitatively adjusted according to participants' energy requirements.

The participants were informed that during the experiment, they had to follow the individually received diet carefully and monitor the weight of the products. This meant eating meals, refraining from snacking on anything extra, and paying attention to the prescribed portions of each. Seafood is a rich source of both omega‐3 fatty acids and TMAO. However, the research was conducted in Poland, where access to fresh and high‐quality seafood is limited and expensive; therefore, it was decided to use only fish in the dietary intervention.

In the representative 2000‐kcal 7‐day meal plan, fish was included in 13 of 21 main meals, with a total planned intake of approximately 2018.5 g/week, corresponding to an average of approximately 288 g/day. The plan comprised cod (900 g/week), fresh salmon (550 g/week), cold‐smoked salmon (50 g/week), pollock (225 g/week), tuna in water (130 g/week), smoked mackerel (103.5 g/week), and smoked trout (60 g/week). Fish products were provided fresh, canned, or smoked according to the meal plan, and daily fish intake ranged from approximately 225 to 350 g/day.

A detailed representative 7‐day 2000‐kcal meal plan, including the exact quantities of individual foods used in each meal, is provided in the Supporting Information (Tables [Link], [Link], [Link], [Link]). Because individual meal plans were scaled according to participants' energy requirements, the quantities shown in the representative plan do not reflect identical food amounts prescribed to all participants; however, the qualitative composition and ketogenic macronutrient profile were maintained across the individualized dietary plans.

With respect to the assessment of the accuracy of self‐reported food types and quantities, each participant had a debriefing interview with the dietitian at the end of the intervention. Additionally, throughout the 14 days, the participants were encouraged to stay in close contact with the dietitian via phone or e‐mail to ask questions or clarify any dietary concerns in real time. The participants ate three meals a day and therefore fasted for at least 10 h per day. A 12‐h fasting period was recommended for the subjects before blood samples were collected for testing.

Nutritional ketosis was objectively confirmed by measurements of ketone bodies in capillary blood and urine. Blood β‐hydroxybutyrate increased from 0.21 ± 0.15 mmol/L at baseline to 2.20 ± 1.19 mmol/L after the 2‐week intervention (*p* < 0.001), while urinary ketone bodies increased from 0.00 ± 0.00 to 4.64 ± 3.24 mmol/L (*p* < 0.001), as previously reported for this cohort [20]. Blood β‐hydroxybutyrate was measured in capillary blood using a FreeStyle Optium Neo glucose and ketone monitoring system with β‐ketone test strips, while urinary acetoacetate was assessed using Keto‐Diastix reagent strips, as described previously [20].

To evaluate adherence to dietary instructions during the 2‐week intervention, compliance was monitored through a similar process: direct communication, consistency in reporting, and final interviews conducted by the supervising dietitian ensured that dietary adherence was maintained and verified.

The contents of choline, betaine, and glycerophosphocholine (GPC), expressed in mg per 100 g of food and based on dietary sources of TMAO, are presented in the Supporting Information (Table S3).

### 2.5 Blood Samples

Capillary blood, plasma, and serum samples were collected from patients before and after KD. All blood samples were obtained from patients seated upright by a qualified physician. The fasting instructions required participants to abstain from food intake for at least 10 h, with a strong recommendation for a 12‐h fasting period prior to sample collection. Blood samples were collected from the antecubital veins. After collection, the samples were centrifuged at 2000× *g* for 10 min at 4°C, and aliquots were stored at −80°C for later analysis.

Plasma TMAO, betaine, choline, GPC, and carnitine levels were measured using a liquid chromatography–mass spectrometry system at the Mass Spectrometry Laboratory, Institute of Biochemistry and Biophysics (Warsaw, Poland), as previously described [22, 23].

The selected glycemic markers in the blood serum were analysed using a magnetic bead‐based assay (Bio‐Rad, Hercules, CA, USA). The Bio‐Plex Pro Human Diabetes Assay (#171A7001M; Bio‐Rad, Hercules, CA, USA) was used to detect fasting levels of C‐peptide, ghrelin, glucagon, insulin, leptin, and resistin. The samples were run in duplicate using a BioPlex 200 instrument (BioRad, Hercules, CA, USA).

Oxidative stress markers were measured in EDTA‐treated plasma samples. The concentration of —SH groups was determined according to Ellman's method [24]. Briefly, the plasma samples were incubated with 5,5′‐dithiobis (2‐nitrobenzoic acid) (DTNB) at 37°C for 20 min, and the absorbance was measured at 412 nm.

The concentrations of total cholesterol (TCh), low‐density lipoprotein (LDL), high‐density lipoprotein (HDL), and triglycerides (TG) in the blood serum were determined using a standard automatic analyser (Cobas 6000; Roche Diagnostics, Mannheim, Germany).

Blood glucose levels in capillary blood were determined using a Freestyle Libre Optium Xido Neo glucometer (Abbott, Abbott Diabetes Care Ltd., Witney, Oxon, UK).

The Homeostatic Model Assessment (HOMA) is a validated mathematical model for estimating insulin resistance from fasting glucose and insulin levels, confirmed against the euglycemic‐hyperinsulinemic clamp [25]. This is the most common method for determining insulin resistance and estimating insulin sensitivity. The updated computer model was used to determine insulin sensitivity (HOMA%S) from paired fasting glucose and insulin levels. The HOMA indices were obtained using the HOMA 2 Calculator software, version 2.2.3, copyrighted by the University of Oxford [26].

### 2.6 Statistical Analyses

All the data are presented as means (*M*) and standard deviations (SD). The normality of the data distribution was assessed using the Shapiro–Wilk test. Depending on the data distribution, for comparison between pre‐ and post‐test, either a paired‐samples *t*‐test or the Wilcoxon signed‐rank test was used. Moreover, effect sizes were calculated as Hedges' *g* for paired *t*‐tests and as r for Wilcoxon tests. Effect sizes were interpreted according to conventional thresholds of 0.2 (small), 0.5 (medium), and 0.8 (large) for Hedges' *g* and 0.1, 0.3, and 0.5 for *r*, respectively.

Prior to regression analysis, correlations between dependent and independent variables were explored to identify candidate variables for inclusion in the regression models of ΔTMAO and ΔHOMA‐%S. Spearman's rank correlation coefficient (*r*_s_) and Pearson's correlation coefficient (*r*_p_) were used for non‐normally and normally distributed variables, respectively.

To examine associations between changes in variables (Δ; post‐test—pre‐test) and two dependent change variables (ΔTMAO and ΔHOMA‐%S), stepwise multiple regression with forward selection was performed. Considering the relatively small sample size and the exploratory nature of the analysis, variables showing significant or near‐significant associations based on unadjusted *p*‐values (*p* < 0.10) with the dependent variables were considered for inclusion in the regression models.

The four assumptions of regression described in the study by Ernst and Albers [27] were met. Each step of the regression analysis was presented in a table including the coefficient of determination (*R* ^2^), change in the coefficient of determination (Δ*R* ^2^), standardized regression coefficients (*β*), *F*‐statistics, and *p* values. Adjusted *R* ^2^ was additionally reported as a more conservative estimate of explained variance in the final regression models. Furthermore, leave‐one‐out cross‐validation (LOOCV) was performed in R to assess the predictive performance of the regression models.

Statistical significance was set at *p* < 0.05 for all analyses. For correlation analyses, associations with 0.05 ≤ *p* < 0.10 were considered near‐significant. To account for multiple comparisons, *p*‐values from correlation analyses and pre‐post comparisons were adjusted using the Benjamini‐Hochberg false discovery rate (FDR) procedure, with FDR correction applied separately within each family of related tests. The same thresholds were applied to FDR‐adjusted *p*‐values, with *p*_adj_ < 0.05 considered statistically significant for both correlation analyses and pre‐post comparisons, whereas 0.05 ≤ *p*_adj_ < 0.10 was considered near‐significant only for correlation analyses. All calculations were performed using TIBCO Statistica (version 13.3; TIBCO Software Inc., 2017).

## 3 Results

A critical element of the present study was the assumption that a diet rich in fish would induce an increase in TMAO concentration in the blood. The results presented in Table 1, confirmed this assumption, as the increase in TMAO was significant and remained significant after FDR correction, with a large effect size (Table 1).

**TABLE 1:** Pre‐ and post‐intervention comparisons following a 2‐week calorie‐restricted, fish‐rich ketogenic dietary intervention in healthy adults.

Variable	Pre‐test		Post‐test		Test's result	*p*	*p*_adj_	Effect size
	*M*	SD	*M*	SD				
TMAO (μM)	3.38	1.93	11.41	9.87	3.18^b^	**0.0015**	**0.0025**	*r* = 0.89
Betaine (μM)	28.21	7.38	47.24	13.67	−7.05^a^	**0.0000**	**0.0000**	*g* = 2.41
Carnitine (μM)	27.76	6.57	22.12	10.89	2.95^a^	**0.0112**	**0.0140**	*g* = 0.80
GPC (μM)	1.11	0.24	0.70	0.20	5.96^a^	**0.0000**	**0.0000**	*g* = 1.60
Choline (μM)	15.96	7.27	13.79	6.14	1.04^b^	0.3003	0.3003	*r* = 0.28
Leptin (ng/mL)	7.32	7.07	3.61	5.32	2.86^b^	**0.0043**	**0.0072**	*r* = 0.76
Resistin (ng/mL)	18.10	5.98	15.16	6.13	2.60^a^	**0.0221**	**0.0221**	*g* = 0.46
C‐peptide (μg/mL)	2.33	1.37	1.30	0.80	4.17^a^	**0.0011**	**0.0055**	*g* = 0.70
Insulin (mIU/L)	15.63	11.06	9.55	7.89	2.73^b^	**0.0063**	**0.0079**	*g* = 0.51
Glucose (mg/dL)	95.07	8.57	87.57	8.39	2.92^b^	**0.0035**	**0.0072**	*g* = 0.82
—SH groups (μmol/mL)	0.46	0.04	0.47	0.05	−0.73^a^	0.4798	0.4798	*g* = 0.23
HOMA‐%S (%)	82.04	58.81	125.50	83.43	1.91^b^	0.0555	0.1110	*r* = 0.51
Total cholesterol (mg/dL)	206.43	37.54	202.29	28.01	0.39^a^	0.7034	0.9379	*g* = 0.10
LDL cholesterol (mg/dL)	125.43	33.64	130.21	26.17	−0.45^a^	0.6573	0.9379	*g* = 0.13
HDL cholesterol (mg/dL)	62.66	12.86	53.31	14.48	5.57^a^	**0.0001**	**0.0004**	*g* = 0.68
Triglycerides (mg/dL)	93.00	31.98	93.50	27.38	−0.06^a^	0.9527	0.9527	*g* = 0.01

*Note:* Statistically significant results (*p* < 0.05 or *p*_adj_ < 0.05) are shown in bold.

Abbreviations: *M*, mean; *p*, *p* values; *p*_adj_, *p* values after FDR correction; SD, standard deviation.

^a^ Paired‐samples *t*‐test.

^b^ Wilcoxon signed‐rank test.

Results showed a significant increase over time in betaine (by 19.03 μM), as well as significant decreases in carnitine (by 5.64 μM) and GPC (by 0.41 μM), all of which remained significant after FDR correction, with large effect sizes (*g* ≥ 0.80). In contrast, choline did not change significantly over time (*p* ≥ 0.05) and remained non‐significant after FDR correction (*p*_adj_ ≥ 0.05), with a small observed effect size (Table 1).

Significant decreases over time were noted in leptin (by 3.71 ng/mL) and glucose (by 7.50 mg/dL), both with large effect sizes, as well as in C‐peptide (by 0.97 μg/mL) and insulin (by 6.08 mIU/L), with medium effect sizes. In contrast, resistin also showed a significant decrease over time (by 2.94 ng/mL), despite the observed effect size being small. All reported pre‐post changes remained significant after FDR correction (*p*_adj_ < 0.05) (Table 1).

Although HOMA‐%S did not change significantly over time, an increase was observed (by 43.46%; *p* = 0.0555), with a large effect size. However, this finding was not supported after FDR correction (*p*_adj_ = 0.1110). In contrast, —SH groups did not change significantly over time (*p* ≥ 0.05) and remained non‐significant after FDR correction (*p*_adj_ ≥ 0.05), with a small observed effect size (Table 1).

A significant decrease in HDL cholesterol was observed over time (−9.35 mg/dL), with a medium effect size, and remained significant after FDR correction (*p*_adj_ < 0.05). In contrast, no significant changes were observed in total cholesterol, LDL cholesterol, or triglycerides (*p* ≥ 0.05 and *p*_adj_ ≥ 0.05), with negligible effect sizes (*g* < 0.20) (Table 1).

Although n‐3 PUFA intake was derived predominantly from plant‐based ALA rather than marine long‐chain n‐3 PUFAs, estimated individual total n‐3 PUFA intake was not significantly associated with the change in HDL cholesterol (ΔHDL‐C; *r*ₛ = 0.22; *p* = 0.456).

Given the significant effects observed above, we further analysed the relationships between changes in TMAO concentration (ΔTMAO) and glycemic control (ΔHOMA‐%S), as well as their associations with changes in other metabolic indicators (Table 2).

**TABLE 2:** Correlation analysis of changes in study variables with ΔTMAO and ΔHOMA‐%S during the 2‐week calorie‐restricted, fish‐rich ketogenic dietary intervention.

Variable	ΔTMAO (*n* = 13)			ΔHOMA‐%S (*n* = 14)		
	*r*_s_	*p*	*p*_adj_	*r*_p_	*p*	*p*_adj_
Δbetaine (μM)	0.47	0.1030	0.4120	−0.24	0.4181	0.5575
Δcartinine (μM)	0.02	0.9574	0.9574	−0.26	0.3742	0.5575
ΔGPC (μM)	0.10	0.7455	0.9574	−0.63	**0.0151**	0.0604
Δcholine (μM)	0.34	0.2629	0.5258	−0.15	0.5999	0.5999
Δleptin (ng/mL)	0.13	0.6676	0.9077	−0.50	0.0667	0.3335
Δresistin (ng/mL)	−0.13	0.6676	0.9077	0.13	0.6574	0.6574
ΔC‐peptide (ng/mL)	0.05	0.8585	0.9077	−0.14	0.6280	0.6574
Δinsulin (mIU/L)	−0.04	0.9077	0.9077	−0.40	0.1552	0.3880
Δglucose (mg/dL)	0.10	0.7393	0.9077	−0.15^s^	0.6184	0.6574
Δ—SH groups (μmol/mL)	0.51	0.0739	0.1478	0.12	0.6785	0.6785
ΔHOMA‐%S (%)	−0.13	0.6808	0.6808	—		
Δtotal cholesterol (mg/dL)	−0.08	0.7956	0.9716	0.39	0.1703	0.1714
ΔLDL cholesterol (mg/dL)	−0.08	0.7890	0.9716	0.39	0.1714	0.1714
ΔHDL cholesterol (mg/dL)	0.01	0.9716	0.9716	−0.40	0.1561	0.1714
Δtriglycerides (mg/dL)	−0.11	0.7208	0.9716	0.43	0.1261	0.1714
Δcarbonyl groups (μmol/mL)	−0.31	0.3848	0.5131	−0.03	0.9245	0.9245
Δbody fat (kg)	0.60	**0.0306**	0.0612	−0.62	**0.0189**	0.0756
Δbody fat (%)	0.66	**0.0132**	0.0528	−0.28	0.3286	0.6572
Δvisceral adipose tissue (g)	0.12	0.7070	0.7070	−0.04	0.9002	0.9245

*Note:* *r*_s_ and ^s^ denote Spearman's rank correlation, *r*_p_—Pearson's correlation. Statistically significant results (*p* < 0.05 or *p*_adj_ < 0.05) are shown in bold, whereas near‐significant results (0.05 ≤ *p* < 0.10 or 0.05 ≤ *p*_adj_ < 0.10) are underlined.

Abbreviations: *p*, *p* values; *p*_adj_, *p* values after FDR correction.

ΔTMAO was significantly positively correlated with Δbody fat expressed as percentage (*r*_s_ = 0.66, *p* = 0.0132) and in kilograms (*r*_s_ = 0.60, *p* = 0.0306) before correction for multiple comparisons. After FDR correction, these associations did not reach statistical significance, but remained near‐significant (*p*_adj_ = 0.0528 and 0.0612, respectively). A near‐significant positive correlation was observed between ΔTMAO and Δ—SH groups (*r*_s_ = 0.51, *p* = 0.0739) before FDR correction; however, this association was no longer near‐significant after correction (*p*_adj_ ≥ 0.10). No other changes in variables were significantly or near‐significantly associated with ΔTMAO either before (*p* ≥ 0.10) or after FDR correction (*p*_adj_ ≥ 0.10) (Table 2).

Meanwhile, ΔHOMA‐%S was significantly negatively correlated with ΔGPC (*r*_p_ = −0.63, *p* = 0.0151) and Δbody fat expressed in kilograms (*r*_p_ = −0.62, *p* = 0.0189) before correction for multiple comparisons. After FDR correction, these associations did not reach statistical significance but remained near‐significant (*p*_adj_ = 0.0604 and 0.0756, respectively). A near‐significant negative correlation was also observed between ΔHOMA‐%S and Δleptin (*r*_p_ = −0.50, *p* = 0.0667) before FDR correction; however, this association was no longer near‐significant after correction (*p*_adj_ ≥ 0.10). No other changes in variables were significantly or near‐significantly associated with ΔHOMA‐%S either before (*p* ≥ 0.10) or after FDR correction (*p*_adj_ ≥ 0.10) (Table 2).

To further investigate the relationships between the analysed variables (changes in blood TMAO concentrations and HOMA‐%S), regression analyses were performed. The results are shown in the table below (Table 3).

**TABLE 3:** Exploratory stepwise multiple regression models for ΔTMAO and ΔHOMA‐%S during the 2‐week calorie‐restricted, fish‐rich ketogenic dietary intervention in healthy adults.

	*R* ^2^	Δ*R* ^2^	*β*	*F*	*p*
**ΔTMAO (*n* = 13)**					
**Step 1**	33.5%			5.53	0.0383
Δbody fat (kg)			0.58		0.0383
**Step 2**	55.0%	21.5%		6.12	0.0184
Δbody fat (kg)			0.50		0.0423
Δ—SH groups (μmol/mL)			0.47		0.0534
**ΔHOMA‐%S (*n* = 14)**					
**Step 1**	40.0%			8.02	0.0151
ΔGPC (μM)			−0.63		0.0151
**Step 2**	57.0%	17.0%		7.28	0.0097
ΔGPC (μM)			−0.47		0.0501
Δbody fat (kg)			−0.44		0.0619

Abbreviations: *β*, standardized regression coefficients; Δ*R* ^2^, change in coefficient of determination; *F*, *F*‐statistic; *p*, *p* values; *R* ^2^, coefficient of determination.

Regression analysis of ΔTMAO included two independent variables and explained 55.0% of the variance in ΔTMAO (*R* ^2^ = 55.0%; adjusted *R* ^2^ = 46.0%; LOOCV *R* ^2^ = 35.2%). Change in body fat accounted for the largest proportion of the explained variance in ΔTMAO (33.5%), while change in —SH groups accounted for an additional 21.5% of the explained variance (Table 3).

Regression analysis of changes in HOMA‐%S also included two independent variables and explained 57.0% of the variance in ΔHOMA‐%S (*R* ^2^ = 57.0%; adjusted *R* ^2^ = 49.1%; LOOCV *R* ^2^ = 33.5%). In this model, change in GPC accounted for 40.0% of the explained variance in ΔHOMA‐%S, whereas change in body fat accounted for an additional 17.0% of the explained variance (Table 3). The lower LOOCV *R* ^2^ values suggest that the predictive performance of both models may be lower than indicated by their original fit.

## 4 Discussion

The present study evaluated the metabolic response to a 2‐week, energy‐restricted (approximately −500 kcal/day), fish‐rich ketogenic diet in healthy adults. The intervention increased circulating TMAO while fasting glucose, insulin, and selected insulin‐related and adipokine biomarkers changed in a favourable direction. Mean oxidative‐stress markers did not change, whereas HDL cholesterol decreased. Because ketogenic macronutrient composition, caloric restriction, high fish intake, weight loss, and reduced adiposity occurred concurrently, their independent contributions cannot be distinguished. In particular, the observed metabolic changes cannot be attributed to the increase in TMAO.

Fish contains preformed TMAO, and fish consumption increases circulating and urinary TMAO concentrations [2, 15, 28, 29]. In the present study, plasma carnitine and GPC decreased, choline remained unchanged, and betaine increased. This pattern is consistent with a substantial contribution of directly ingested fish‐derived TMAO, although endogenous microbial production was not measured and cannot be excluded. The intervention also provided approximately twice the total choline intake previously reported in healthy Polish adults, whereas betaine intake was lower [30]. However, the divergent changes in circulating precursors indicate that dietary intake alone may not predict their plasma concentrations.

Circulating TMAO has been associated with diabetes in observational research. A dose–response meta‐analysis reported 54% higher odds of prevalent diabetes per 5 μmol/L increment in TMAO [8]. However, prospective studies have reported no association with incident diabetes in middle‐aged and older adults [31, 32], and an inverse association was observed in an older Mediterranean population [33]. These differences may reflect variation in study populations, dietary sources of TMAO, metabolic health, and study design. In the present intervention, the marked rise in TMAO was not accompanied by deterioration in glycemic control; instead, fasting glucose, insulin, and selected related biomarkers improved. This finding neither contradicts long‐term observational associations nor demonstrates a beneficial effect of TMAO. It indicates only that a short‐term increase in TMAO induced by a fish‐rich, energy‐restricted ketogenic diet can occur alongside favourable glycemic changes in healthy adults.

Plasma betaine also increased during the intervention. Higher betaine concentrations have been associated with lower diabetes risk in several cohorts [33, 34, 35], although not consistently [32]. In the present study, the change in betaine was not directly associated with the change in insulin sensitivity. Moreover, changes in GPC and body fat, rather than TMAO, independently predicted ΔHOMA‐%S. These exploratory findings are compatible with the possibility that changes in insulin‐related biomarkers were driven mainly by the negative energy balance, altered macronutrient intake, and loss of adiposity rather than by TMAO.

Additionally, there is great interest in the correlation between HOMA and adipokines (especially leptin and resistin). Moderate to high physical activity in people with obesity could lower obesity indicators (such as adipokines, insulin resistance, glucose levels, and HOMA) [35]. In addition, energy‐restrictive KD in obese individuals may influence inflammation (generated by fat tissue) and decrease diabetes‐associated parameters or the status of adipokines (leptin and resistin) [36].

Despite reports that TMAO may exert pro‐oxidative effects [37, 38], none of the measured oxidative‐stress markers changed significantly after the intervention. Mean plasma —SH concentrations remained similar to baseline values. The change in TMAO showed a positive, but nonsignificant, association with the change in —SH groups, and Δ—SH was a borderline positive predictor of ΔTMAO in hierarchical regression. Given the absence of group‐level changes, the small sample, and *p* values above 0.05, these results do not establish a redox effect of TMAO. They may indicate a compensatory thiol response or may represent a chance finding and should therefore be treated as hypothesis‐generating. This cautious interpretation is also warranted because plasma —SH groups used here are not directly comparable with erythrocyte —SH groups previously reported to increase after a 2‐week ketogenic diet rich in animal‐derived fats [39].

Interestingly, HDL cholesterol decreased significantly during the intervention, despite favourable changes in several other metabolic parameters. This finding was unexpected, as ketogenic diets have generally been reported to increase HDL cholesterol or have no effect on it [40, 41, 42, 43, 44]. Moreover, in our previous 2‐week ketogenic intervention rich in saturated animal fats, HDL cholesterol increased [45].

The present intervention was characterized by a very high intake of n‐3 PUFAs, predominantly in the form of ALA supplied by flaxseed oil. However, individual total n‐3 PUFA intake was not significantly associated with ΔHDL. Thus, the available data do not support n‐3 PUFA intake as the primary explanation for the observed reduction, while the specific contribution of marine‐derived n‐3 PUFAs cannot be determined. An alternative explanation may involve the negative energy balance and the associated reductions in body weight and adiposity, including a notable decrease in visceral adipose tissue [20]. Because caloric restriction, weight loss, the ketogenic macronutrient composition, and the specific dietary fatty‐acid profile occurred simultaneously, their individual contributions to the reduction in HDL cholesterol cannot be distinguished. Furthermore, HDL and LDL subfractions, particle size and number, and HDL functionality were not assessed. Consequently, the clinical and mechanistic significance of the reduction in HDL cholesterol remains uncertain. Further controlled studies incorporating detailed lipoprotein profiling are required to clarify the mechanisms underlying this finding.

The study has several limitations. First, its short duration, small sample (9 men and 5 women), and single‐arm pre–post design limit both statistical power and generalizability and prevent separation of the effects of caloric restriction, ketosis, fish intake, and changes in body composition. The sample was also insufficient for reliable sex‐stratified analyses. Second, numerous exploratory comparisons were performed without formal correction for multiplicity, increasing the risk of Type I error. Predictor selection for the regression models was based on unadjusted correlation screening in the same dataset used for model estimation, which increases the risk of overfitting; leave‐one‐out cross‐validation does not eliminate this bias. Accordingly, the regression results should be viewed as exploratory. Third, adherence was assessed mainly from dietary records, participant reports, and contact with a dietitian, although the increase in circulating ketone bodies provides objective evidence that ketosis was achieved. Finally, the absence of detailed lipoprotein profiling limits interpretation of the HDL response.

A graphical summary of the key metabolic, lipid, and glycemic changes observed during the 2‐week, calorie‐restricted, fish‐rich ketogenic dietary intervention in healthy adults is presented in Figure 2.

**FIGURE 2:**
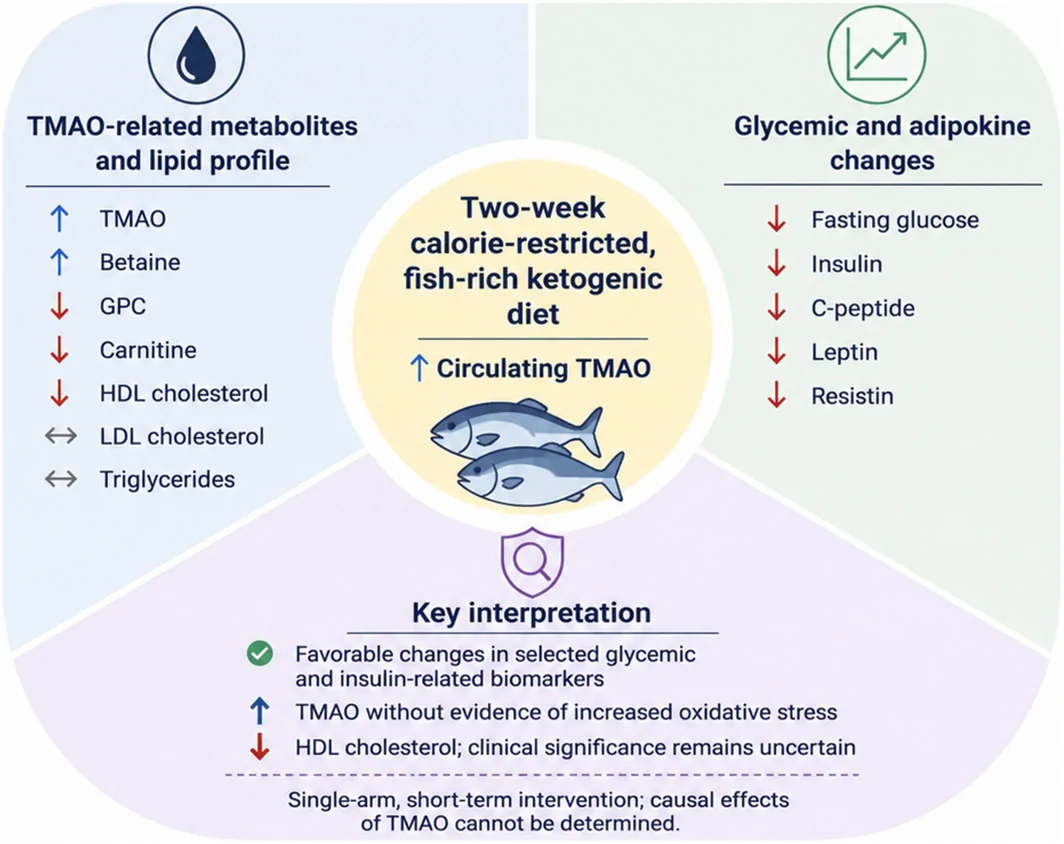
Summary of changes during the 2‐week, energy‐restricted, fish‐rich ketogenic diet. Arrows indicate significant increases (↑), decreases (↓), or no significant changes (↔) in circulating TMAO and related metabolites, lipid‐profile measures, and selected glycemic and adipokine biomarkers. The arrows describe direction only and do not imply clinical benefit, harm, or causality. Because this was a short, single‐arm pre–post study, the effects of caloric restriction, ketogenic macronutrient composition, fish intake, weight and fat loss, and increased TMAO cannot be separated.

Taking the above into account, we decided to investigate the interactions between TMAO and its precursors in its synthesis and the studied indicators of metabolic and glycemic health.

The results presented here therefore suggest a possible dual role for TMAO. In general, high TMAO concentrations are not associated with negative disturbances in the glycemic and metabolic health indicators studied. However, it cannot be ruled out that in patients with chronic inflammation and oxidative stress, TMAO may play a pro‐oxidant role.

Nonetheless, future investigations employing a longer‐duration dietary intervention and larger sample sizes will be required to more clearly delineate the respective contributions of caloric restriction and macronutrient composition to the observed metabolic outcomes. Future studies should include a non‐ketogenic control group to distinguish the effects of caloric restriction from those of ketogenic dietary composition. Further investigation into the effects of this diet among individuals with Type 2 diabetes and chronic low‐grade inflammation would also be of considerable interest.

## 5 Conclusion

In conclusion, a 2‐week, energy‐restricted, fish‐rich ketogenic diet increased circulating TMAO but did not worsen the measured glycemic or oxidative‐stress markers in healthy adults; selected insulin‐related biomarkers and body‐composition measures improved, whereas HDL cholesterol decreased. These concurrent changes should not be interpreted as effects of TMAO itself. Larger, longer, controlled trials with a non‐ketogenic energy‐restricted comparator, detailed lipoprotein profiling, and prespecified analyses are needed to separate the effects of energy restriction, ketogenic macronutrient composition, fish intake, and weight loss. Studies in populations with Type 2 diabetes or chronic low‐grade inflammation may clarify whether the metabolic context modifies responses to diet‐induced increases in TMAO.

## Author Contributions

**Katarzyna Siedzik:** conceptualization, investigation, funding acquisition, writing – original draft, methodology, data curation, formal analysis, project administration, supervision, validation, visualization, writing – review and editing. **Kamil Góral:** data curation, investigation. **Damian Flis:** investigation, writing – review and editing. **Maciej Jaguszewski:** data curation, formal analysis. **Robert A. Olek:** conceptualization, data curation, investigation, supervision, writing – original draft, writing – review and editing. **Wiesław Ziółkowski:** conceptualization, formal analysis, methodology, supervision, visualization, writing – original draft, writing – review and editing.

## Funding

This study was supported by grants from the Ministry of Science and Higher Education—Development of Young Researchers (to K.S. in the 2020/2021, 2021/2022 (16/NSN/W/2022) and 2022/2023 (6/NSN/W/2023) academic years).

## Ethics Statement

The study was conducted in accordance with the standards for human research as outlined in the Declaration of Helsinki. The study protocol was approved by the Independent Bioethics Committee for Scientific Research, District Medical Chamber in Gdansk (KB 27‐19) and was registered in the clinicaltrials.gov registry (NCT05558488). All participants were informed about the basic principles of the KD, including allowed and prohibited products, risks, and expected outcomes before starting the experimental procedures. Written informed consent for participation was obtained.

## Consent

All the authors agree to publish this work.

## Conflicts of Interest

The authors declare no conflicts of interest.

## Supporting information


**Table S1:** Representative 7‐day 2000‐kcal ketogenic dietary plan used in the intervention. The following meal plan represents the 2000‐kcal version of the ketogenic dietary intervention. Individual dietary plans were based on the same qualitative food composition but were quantitatively adjusted according to each participant's estimated energy requirement while maintaining an approximately 500 kcal/day energy deficit and the predefined ketogenic macronutrient composition. The quantities presented below therefore illustrate the reference 2000‐kcal diet and should not be interpreted as identical food amounts prescribed to every participant.

**Table S2:** Fish quantity and frequency in the representative 2000‐kcal dietary plan.

**Table S3:** Choline, betaine, and glycerophosphocholine contents of foods included in the dietary intervention based on the USDA Database for the Choline Content of Common Foods, Release 2 (2008).

**Table S4:** Calculated daily intake of total choline, betaine, and glycerophosphocholine in the representative 2000‐kcal dietary plan.

## Data Availability

The datasets used and analysed during the current study are available from the corresponding author.
