# Supplementary material for: Metabolic Changes During a Calorie‐Restricted, Fish‐Rich Ketogenic Diet Associated With Increased Circulating TMAO in Healthy Adults
Source: Endocrinol Diabetes Metab. 2026 Oct 16;9(6):e70360. doi: 10.1002/edm2.70360 (PMC13673408; doi:10.1002/edm2.70360)
Supplement: Supplementary file 1 [file EDM2-9-e70360-s001.docx]

**Table S2**

**Fish quantity and frequency in the representative 2000-kcal dietary plan**

| **Fish product** | **Weekly amount (g)** | **Number of fish-containing meals** |
| --- | --- | --- |
| Fresh cod | 900 | 5 |
| Fresh salmon | 550 | 3 |
| Cold-smoked salmon | 50 | 1 |
| Fresh pollock | 225 | 1 |
| Tuna in water | 130 | 1 |
| Smoked mackerel | 103.5 | 1 |
| Smoked trout | 60 | 1 |
| **Total fish intake** | **2018.5 g/week** | **13 meals/week** |
| **Mean fish intake** | **288.4 g/day** | — |

The values presented refer to the 2000-kcal reference version of the intervention. Fish was included on all seven days of the representative dietary plan and in 13 of the 21 main meals. Daily fish intake ranged from 225 to 350 g/day.
