# Supplementary material for: Metabolic Changes During a Calorie‐Restricted, Fish‐Rich Ketogenic Diet Associated With Increased Circulating TMAO in Healthy Adults
Source: Endocrinol Diabetes Metab. 2026 Oct 16;9(6):e70360. doi: 10.1002/edm2.70360 (PMC13673408; doi:10.1002/edm2.70360)
Supplement: Supplementary file 2 [file EDM2-9-e70360-s002.docx]

**Table S1**

**Representative 7-day 2000-kcal ketogenic dietary plan used in the intervention**

The 2000-kcal version of the diet provided approximately 2001.6 kcal/day, including 101.4 g protein, 170.2 g fat, and 20.5 g total carbohydrates, corresponding to approximately 20.2%, 76.4%, and 3.4% of total energy intake, respectively. Digestible carbohydrate intake was approximately 13.1 g/day, and dietary fiber intake was approximately 7.4 g/day.

| **Day** | **Meal** | **Food items and amounts** | **Fish intake (g)** |
| --- | --- | --- | --- |
| **1** | Breakfast | Whole eggs, 236 g; romaine lettuce, 37.5 g; dill pickles, 50 g; flaxseed oil, 32 g | **–** |
|  | Lunch | Fresh salmon, 200 g; tomatoes, 54 g; carrots, 52.5 g; frozen cauliflower, 90 g; flaxseed oil, 40 g | **200** |
|  | Dinner | Avocado, 70 g; dill pickles, 50 g; tuna in water, 130 g; flaxseed oil, 40 g | **130** |
| **Daily total** | | | **330** |
| **2** | Breakfast | Tomatoes, 108 g; whole eggs, 236 g; romaine lettuce, 50 g; dill pickles, 50 g; flaxseed oil, 40 g | **–** |
|  | Lunch | Fresh cod, 150 g; curry powder, 2.5 g; dill pickles, 50 g; romaine lettuce, 25 g; mozzarella, 60 g; tomatoes, 81 g; flaxseed oil, 40 g; spinach, 30 g | **150** |
|  | Dinner | Smoked mackerel, 103.5 g; fresh parsley, 18 g; carrots, 26.3 g; spinach, 30 g; flaxseed oil, 40 g | **103.5** |
| **Daily total** | | | **253.5** |
| **3** | Breakfast | Tomatoes, 54 g; whole eggs, 236 g; romaine lettuce, 25 g; dill pickles, 50 g; flaxseed oil, 40 g | **–** |
|  | Lunch | Fresh pollock, 225 g; spinach, 30 g; spring onion, 20 g; feta cheese, 100 g; tomatoes, 54 g; flaxseed oil, 40 g | **225** |
|  | Dinner | Fresh cod, 100 g; carrots, 52.5 g; parsley root, 13.5 g; leek, 59 g; flaxseed oil, 48 g | **100** |
| **Daily total** | | | **325** |
| **4** | Breakfast | Tomatoes, 108 g; whole eggs, 236 g; romaine lettuce, 25 g; dill pickles, 50 g; flaxseed oil, 32 g; semi-fat curd cheese (twaróg), 25 g | **–** |
|  | Lunch | Fresh cod, 225 g; flaxseed oil, 40 g; coconut milk, 60 g; red sweet pepper, 37.5 g; tahini, 24 g | **225** |
|  | Dinner | Cold-smoked salmon, 50 g; avocado, 70 g; fresh parsley, 18 g; spinach, 10 g; semi-fat curd cheese (twaróg), 25 g; flaxseed oil, 32 g | **50** |
| **Daily total** | | | **275** |
| **5** | Breakfast | Tomatoes, 108 g; whole eggs, 177 g; romaine lettuce, 50 g; dill pickles, 50 g; flaxseed oil, 32 g | **–** |
|  | Lunch | Onion, 35 g; tomatoes, 108 g; spinach, 30 g; fresh cod, 200 g; flaxseed oil, 40 g | **200** |
|  | Dinner | Fresh salmon, 150 g; tomatoes, 81 g; dill pickles, 50 g; feta cheese, 80 g; spinach, 30 g; flaxseed oil, 40 g | **150** |
| **Daily total** | | | **350** |
| **6** | Breakfast | Tomatoes, 108 g; whole eggs, 177 g; romaine lettuce, 50 g; dill pickles, 50 g; flaxseed oil, 28 g | **–** |
|  | Lunch | Red sweet pepper, 37.5 g; onion, 17.5 g; mustard, 5 g; Herbes de Provence, 1 g; fresh salmon, 200 g; flaxseed oil, 40 g | **200** |
|  | Dinner | Tomatoes, 108 g; whole eggs, 177 g; smoked trout, 60 g; red sweet pepper, 37.5 g; flaxseed oil, 32 g | **60** |
| **Daily total** | | | **260** |
| **7** | Breakfast | Tomatoes, 54 g; spinach, 30 g; flaxseed oil, 32 g; whole eggs, 236 g; chives, 8 g | **–** |
|  | Lunch | Herbes de Provence, 2 g; fresh cod, 225 g; flaxseed oil, 48 g; tomatoes, 108 g; onion, 17.5 g | **225** |
|  | Dinner | Frozen cauliflower, 112.5 g; almond flakes, 30 g; feta cheese, 100 g; flaxseed oil, 32 g | **–** |
| **Daily total** | | | **225** |
| **7-day total** | | | **2018.5** |
| **Mean/day** | | | **288.4** |
