# Supplementary material for: Metabolic Changes During a Calorie‐Restricted, Fish‐Rich Ketogenic Diet Associated With Increased Circulating TMAO in Healthy Adults
Source: Endocrinol Diabetes Metab. 2026 Oct 16;9(6):e70360. doi: 10.1002/edm2.70360 (PMC13673408; doi:10.1002/edm2.70360)
Supplement: Supplementary file 3 [file EDM2-9-e70360-s003.docx]

**Table S4**

**Calculated daily intake of total choline, betaine, and glycerophosphocholine in the representative 2000-kcal dietary plan.**

| **Day** | **Total choline (mg/day)** | **Betaine (mg/day)** | **GPC (mg/day)** |
| --- | --- | --- | --- |
| Day 1 | 870.3 | 9.5 | 92.8 |
| Day 2 | 883.0 | 88.1 | 82.0 |
| Day 3 | 906.9 | 67.3 | 102.6 |
| Day 4 | 872.9 | 36.5 | 96.0 |
| Day 5 | 803.9 | 90.2 | 125.6 |
| Day 6 | 1149.1 | 7.8 | 112.8 |
| Day 7 | 873.4 | 57.7 | 73.3 |
| **Weekly total** | **6359.5** | **357.1** | **685.1** |
| **Mean daily intake** | **908.5** | **51.0** | **97.9** |

**Note:** Values were calculated for the representative 2000-kcal dietary plan using the food quantities presented in Table S1 and the USDA food-composition values presented in Table S3. Individual dietary plans followed the same qualitative composition but were quantitatively adjusted according to participants’ estimated energy requirements; therefore, these values should not be interpreted as identical intake values for all participants.
