# Supplementary material for: Metabolic Changes During a Calorie‐Restricted, Fish‐Rich Ketogenic Diet Associated With Increased Circulating TMAO in Healthy Adults
Source: Endocrinol Diabetes Metab. 2026 Oct 16;9(6):e70360. doi: 10.1002/edm2.70360 (PMC13673408; doi:10.1002/edm2.70360)
Supplement: Supplementary file 4 [file EDM2-9-e70360-s004.docx]

**Table S3**

**Choline, betaine, and glycerophosphocholine contents of foods included in the dietary intervention based on the USDA Database for the Choline Content of Common Foods, Release 2 (2008)**

| **Food item in dietary plan** | **USDA food description used** | **USDA NDB No.** | **Match** | **Betaine (mg/100 g)** | **Total choline (mg/100 g)** | **GPC (mg/100 g)** | **Rationale** |
| --- | --- | --- | --- | --- | --- | --- | --- |
| Whole chicken eggs | Egg, whole, raw, fresh | 01123 | Exact | 0.6 | 250 | 0.6 | Direct match to whole raw egg. The corrected value was used instead of the egg-white value erroneously assigned to whole egg in the original Table S1. |
| Romaine lettuce | Lettuce, cos or romaine, raw | 11251 | Exact | 0.1 | 9.9 | 0.0 | Direct match. |
| Dill pickles | Pickles, cucumber, dill or kosher dill | 11937 | Exact | 0.0 | 3.4 | 0.3 | Direct match to dill-pickled cucumber. |
| Flaxseed oil | Oil, olive, salad or cooking | 04053 | Proxy – oil type | 0.1 | 0.3 | 0.3 | Flaxseed oil was not represented in the USDA 2008 choline database; olive oil was used as an oil-based proxy for these compounds. |
| Fresh salmon | Fish, salmon, Atlantic, farmed, cooked, dry heat | 15237 | Proxy – preparation/source | 1.8 | 91 | 41 | Closest available entry for cooked salmon; the menu did not specify wild/farmed origin. |
| Red tomatoes | Tomatoes, red, ripe, raw, year-round average | 11529 | Exact | 0.1 | 6.7 | 0.0 | Direct match. |
| Carrots | Carrots, raw | 11124 | Exact | 0.4 | 8.8 | 0.0 | Direct match. |
| Frozen cauliflower | Cauliflower, cooked, boiled, drained, without salt | 11136 | Proxy – preparation | 0.1 | 39 | 0.7 | Same vegetable; USDA entry refers to cooked cauliflower rather than specifically frozen cauliflower. |
| Avocado | Avocados, raw, California | 09038 | Exact / cultivar assumption | 0.7 | 14 | 0.7 | Same food and preparation state; California avocado was the available USDA entry. |
| Tuna in water | Fish, tuna, light, canned in water, drained solids | 15121 | Exact | 2.7 | 29 | 5.9 | Direct match. |
| Fresh cod | Fish, cod, Atlantic, cooked, dry heat | 15016 | Proxy – preparation | 9.7 | 84 | 30 | Same fish species; meal preparation differed from the USDA dry-heat entry. |
| Curry powder | Spices, curry powder | 02015 | Exact | 29 | 64 | 1.1 | Direct match. |
| Mozzarella | Cheese, mozzarella, part skim milk, low moisture | 01029 | Exact / product-form assumption | 0.7 | 14 | 2.7 | Same cheese type; exact moisture/fat specification was not provided in the meal plan. |
| Spinach | Spinach, frozen, chopped or leaf, cooked, boiled, drained, without salt | 11464 | Proxy – preparation | 110 | 25 | 0.0 | USDA entry for cooked frozen spinach was used because the meal plan did not specify the preparation form. |
| Smoked mackerel | Fish, salmon, king, kippered, raw | 98101 | Proxy – species | 4.3 | 110 | 32 | No appropriate mackerel entry was available; another smoked/kippered oily fish was used as proxy. |
| Fresh parsley | Basil, fresh | 02044 | Proxy – fresh herb | 0.4 | 11 | 0.7 | Fresh parsley was unavailable with all three compounds; fresh basil was used as a fresh-herb proxy. |
| Fresh pollock | Fish, cod, Atlantic, cooked, dry heat | 15016 | Proxy – closely related lean fish | 9.7 | 84 | 30 | Pollock was unavailable; cod was selected as a closely related lean gadid fish. |
| Spring onion / scallion | Onions, raw | 11282 | Proxy – related *Allium* | 0.1 | 6.1 | 0.6 | No corresponding scallion entry was available with all required compounds. |
| Feta cheese | Cheese, mozzarella, part skim milk, low moisture | 01029 | Proxy – cheese type | 0.7 | 14 | 2.7 | Feta was unavailable; mozzarella was used as the closest available cheese proxy. |
| Parsley root | Carrots, raw | 11124 | Proxy – root vegetable | 0.4 | 8.8 | 0.0 | Parsley root was unavailable; carrot was used as a root-vegetable proxy. |
| Leek | Onions, raw | 11282 | Proxy – related *Allium* | 0.1 | 6.1 | 0.6 | Leek was unavailable; raw onion was used as an *Allium*-family proxy. |
| Semi-fat curd cheese (twaróg) | Cheese, cottage, creamed, large or small curd | 01012 | Proxy – dairy product | 0.7 | 18 | 8.4 | Polish semi-fat twaróg had no direct USDA equivalent; cottage cheese was used as the closest available dairy product. |
| Coconut milk | Nuts, coconut milk, canned | 12118 | Proxy – preparation | 0.0 | 8.5 | 0.6 | Same basic food matrix; USDA entry was canned whereas the menu specified homemade coconut milk. |
| Red sweet pepper | Peppers, sweet, red, raw | 11821 | Exact | 0.1 | 5.6 | 0.5 | Direct match. |
| Tahini | Seeds, sesame seed kernels, dried, decorticated | 12201 | Proxy – processed form | 0.7 | 26 | 3.1 | Tahini itself was unavailable; decorticated sesame kernels were used as its principal ingredient. |
| Cold-smoked salmon | Fish, salmon, Atlantic, farmed, cooked, dry heat | 15237 | Proxy – processing | 1.8 | 91 | 41 | Same fish species was prioritized, although the USDA preparation differed from cold smoking. |
| Onion | Onions, raw | 11282 | Exact | 0.1 | 6.1 | 0.6 | Direct match. |
| Prepared mustard | Mustard, prepared, yellow | 02046 | Exact | 0.2 | 22 | 2.4 | Direct match. |
| Herbes de Provence | Spices, oregano, dried | 02027 | Proxy – herb mixture | 9.8 | 32 | 1.6 | The herb mixture had no direct entry; dried oregano was used as a representative Mediterranean herb. |
| Smoked trout | Fish, steelhead trout, boiled, canned | 35181 | Proxy – processing | 2.5 | 90 | 46 | Closely related fish entry used because a corresponding smoked-trout entry was unavailable. |
| Chives | Onions, raw | 11282 | Proxy – related *Allium* | 0.1 | 6.1 | 0.6 | Chives were unavailable; onion was used as an *Allium*-family proxy. |
| Almond flakes | Nuts, almonds | 12061 | Exact / physical form | 0.5 | 52 | 1.2 | Flaking does not change the underlying food identity; plain almonds were used. |

**Note**: Food composition values were obtained from the *USDA Database for the Choline Content of Common Foods, Release 2 (2008)*. An Exact match indicates that the corresponding food was available in the database, whereas Proxy indicates that the closest biologically and/or technologically comparable food was used when an exact entry containing values for betaine, total choline, and GPC was unavailable. Total choline includes GPC; therefore, GPC was reported as a separate choline fraction and was not added to total choline. Betaine was considered separately.
